# Supplementary figures and images for: Opposing chemosensory functions of closely related gustatory receptors
Source: eLife. 2023 Dec 7;12:RP89795. doi: 10.7554/eLife.89795 (PMC10703443; doi:10.7554/eLife.89795)

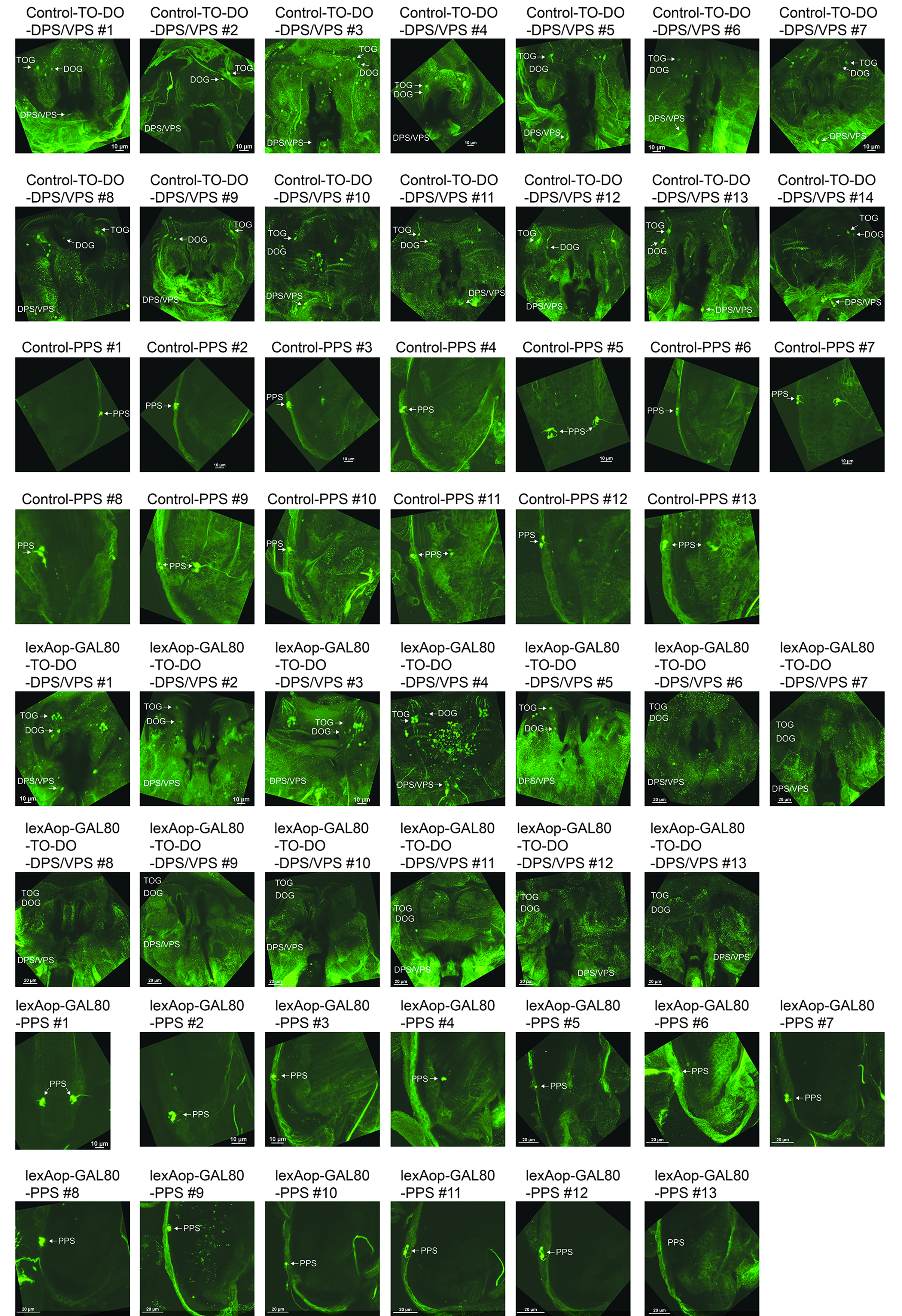

Supplement: Figure 2—figure supplement 1—source data 1. — (B) Quantification of GFP positive GRNs recorded from images of Gr28a GRNs expressing UAS-GCaMP6m (A). [file elife-89795-fig2-figsupp1-data1.zip › Figure 2figure supplement 1Source Data 1.jpg]
